# Effects of Collagen and Collagen Hydrolysate from Jellyfish Umbrella on Histological and Immunity Changes of Mice Photoaging

**DOI:** 10.3390/nu5010223

**Published:** 2013-01-17

**Authors:** Jian Fan, Yongliang Zhuang, Bafang Li

**Affiliations:** 1 College of Chemistry and Engineering, Kunming University of Science and Technology, Kunming, 650500, Yunnan, China; E-Mail: 27664975@qq.com; 2 Department of Food Chemistry and Nutrition, College of Food Science and Engineering, Ocean University of China, Qingdao 266003, Shandong, China; E-Mail: qdli1999@yahoo.com.cn

**Keywords:** jellyfish, collagen, photoaging, histological analysis, immunity

## Abstract

Jellyfish collagen (JC) was extracted from jellyfish umbrella and hydrolyzed to prepare jellyfish collagen hydrolysate (JCH). The effects of JC and JCH on UV-induced skin damage of mice were evaluated by the skin moisture, microscopic analyses of skin and immunity indexes. The skin moisture analyses showed that moisture retention ability of UV-induced mice skin was increased by JC and JCH. Further histological analysis showed that JC and JCH could repair the endogenous collagen and elastin protein fibers, and could maintain the natural ratio of type I to type III collagen. The immunity indexes showed that JC and JCH play a role in enhancing immunity of photoaging mice *in vivo*. JCH showed much higher protective ability than JC. These results suggest that JCH as a potential novel antiphotoaging agent from natural resources.

## 1. Introduction

UV radiation is the main cause of skin damage, which includes photoaging, local and systemic immunosuppression, and photocarcinogenesis [1]. UV radiation interacts with cellular chromatophores and photosensitisers, resulting in the generation of reactive oxygen species (ROS), DNA damage and activation of signaling pathways related to cell/tissue growth, differentiation, senescence and connective tissue degradation [2]. The discovery and development of novel protective and therapeutic agents against UV-induced photodamage is critical for preventing and repairing the damaging effects of UVR in human skin.

Antioxidants have been proven to be effective in the protection of skin against UV-induced damage in recent years. Some dietary antioxidants have shown potential chemoprophylactic activities, including chlamys farreri peptide [3], ferulic acid [4], and cod polypeptide [5]. 

Collagen is another newly founded antioxidant resource. It is a very important raw material in medicine and the food industry. Collagen hydrolysates, which are generally obtained by enzymatic proteolysis from collagen, have exhibited numerous bioactivities, including antioxidant activity, mineral binding capacity, antihypertensive activity, lipid-lowering effect, immunomodulatory activity, *etc.* [6]. The properties of collagen hydrolysates such as good bioactivity, biocompatibility, and penetrability as well as, reparative ability to skin, and less irritation make it a popular regent for developing skin care products [7]. 

In our previous study, jellyfish collagen (JC) and jellyfish collagen hydrolysate (JCH) showed protective effects on the activities of antioxidant enzymes and the content of glutathione in skin photoaging [7]. Furthermore, JC and JCH could protect skin lipid and hydroxyproline contents from the UV radiation damages [7]. To further verify the preventive effect of JC and JCH on skin damage induced by UV light, a histological study was used to illustrate their effect on skin structure, endogenous collagen, elastin protein fibers, and the ratio of type III to type I collagen. In addition, the thymus index (TI) and spleen index (SI) were evaluated to explore the effects of JC and JCH on immunity of mice photoaging *in vivo*.

## 2. Materials and Methods

### 2.1. Animals

ICR male mice (20–22 g), aged about six weeks, were purchased from the Beijing Vital River Experiment Animal Technology Limited Company (Beijing, China). All animal experiments were carried out in accordance with standard guidelines for the care of animals, which were approved by Welfare Committee of the Centre of Experimental Animal (Qingdao, China).

### 2.2. Preparation of JC and JCH

JC was extracted as described previously [8]. A progressive hydrolysis procedure was used to prepare JCH. Firstly, 1% (w/v) JC in water was hydrolyzed by treating with a 2% properase E at pH 8.0, 50 °C for 3 h. Then the pH was reduced to 7.5 and the preparation was continuously hydrolyzed by addition of 1% trypsin at 45 °C for 3 h. The resulting solution was centrifuged at 8000× *g* for 10 min. JC and JCH fractions were freeze-dried and used for the following experiments.

### 2.3. Experimental Design

The mice were fed ad libitum and housed under conventional conditions at a controlled temperature (23 ± 2 °C) humidity (55% ± 10%) and light (12 h light/12 h darkness, without any ultraviolet emission). After one week of acclimatization to the homecage, the mouse back was denuded using sulfureted sodium (8%, Jinshan Co. Ltd., Shanghai, China) over the depilation area of 4 cm^2^, and the animals were randomly divided into the following six groups (eight mice in each group), including NC: normal group; MC: model group; JC-1: at dose 50 mg/kg·day bw JC; JC-2: at dose 200 mg/kg·day bw JC; JCH-1: at dose 50 mg/kg·day bw JCH; JCH-2: at dose 200 mg/kg·day bw JCH by gavage. The mice in NC and MC groups were given normal saline. All mice, except the normal group, were irradiated with the same UV source.

### 2.4. UV Irradiation

Toshiba FL20SE lamps were used as a UV source without any filtering. The distance from the lamps to the animals’ back was 30 cm. The minimal erythemal dose (MED) was preliminarily measured with a UV-radiometer-305, and 290 mJ/cm^2^ of UVA and 28 mJ/cm^2^ of UVB were assembled 1 MED in this study. Mice were irradiated three times weekly (Monday, Wednesday and Friday). Then, intensities of UV were increased by 1 MED per week until week 5, and then maintained at 4 MED up to the 10th week, yielding a total dose of 26.76 and 2.55 J/cm^2^ of UVA and UVB, respectively.

### 2.5. Measurement of Water Content

The moisture of the skin was measured by drying the samples in an oven at 105 °C for 4 h, as described by GB/T5009.3-2010, China [9].

### 2.6. Histological Analysis

Skin specimens were taken for histochemical investigation 24 h after the final irradiation. Mouse skin samples were fixed in 4% buffered neutral formalin solution for 24 h, and embedded in paraffin. Serial sections (7 μm) were mounted onto silane-coated slides and stained with H & E, VG, Verhoeff-van Gieson, and picrosirius red staining. The images were recorded using the Olympus DP70 Digital Camera System at 200× magnifications.

### 2.7. Measurement of Spleen Index and Thymus Index

The animals were weighed and executed by cervical dislocation. Spleen and thymus were excised from the animal and weighed immediately. The thymus and spleen index was calculated according to the following equation [2]: thymus or spleen index (mg/g) = (weight of thymus or spleen)/body weight.

### 2.8. Statistical Analysis

All data were analyzed by one-way analysis of variance (ANOVA) using SPSS (version 11.0, Chicago, IL, USA) and were displayed as mean ± SD. A *p* value of *<*0.05 was taken as the level of statistical significance.

## 3. Results and Discussion

### 3.1. Characterizations of JC and JCH

JC and JCH were prepared as described in materials and methods [7]. The molecular weight (MW) and amino acids compositions of JC and JCH were determined. The results showed that JC was mainly composed of α1 chains (about 105 kDa), which was similar to that of type I collagen of bovine. The MW of JCH was less than 5 kDa. So, the MW of JCH was much lower than that of JC, while, the antioxidant activity of JCH was significantly higher than that of JC. JC and JCH exhibited similar amino acid compositions, and both were rich in Gly, Pro, Glu, Asp, Ala, and total hydrophobic amino acids [7]. 

### 3.2. Determination of Water Content

The water content of the skin is greatly influenced by ground substances, which may be responsible for wrinkling and laxity of the skin accompanying cutaneous ageing [10]. Excessive UV exposure of skin causes abnormal skin water loss. In the NC mice, the water content of the skin was 72.93% (Table 1), which was significantly decreased to 63.38% in the MC group (*p* < 0.05). However, in group JC-1 and JCH-1, 26.60% and 41.15% of water was rescued (Table 1). This result showed that JC and JCH prevented the loss of water contents and this effect is dose-dependent. Interestingly, JCH showed much stronger effect than JC at the same dose. Compared with JC-2 mice, the water content in mice treated with JCH-2 was significantly increased (*p* < 0.05).

**Table 1:** Effects of JC and JCH on water contents of photoaging mice skin (*n* = 8).

Group	Water Content (%)
NC	72.93 ± 1.64 a
MC	63.38 ± 3.99 c
JC-1	65.92 ± 2.32 bc
JC-1	67.35 ± 1.79 b
JCH-1	67.31 ± 2.68 b
JCH-2	69.54 ± 0.87 a

Different letter indicates significant differences (*p* < 0.05).

### 3.3. The Effects of JC and JCH on Histological Changes of Photoaging Skin

#### 3.3.1. The Effects of JC and JCH on Morphology of Photoaging Skin

**Figure 1:**
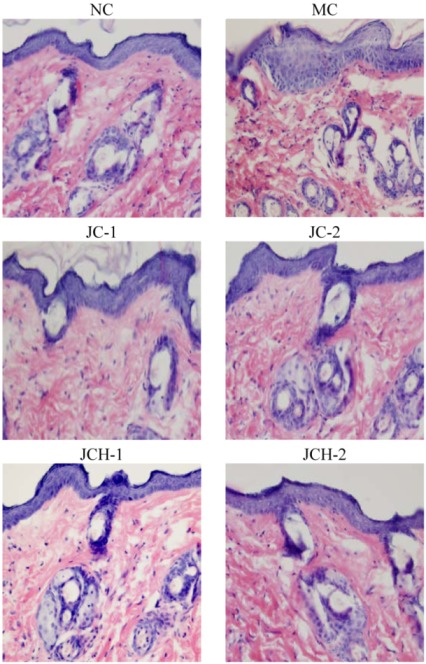
The effect of JC and JCH on morphology of photoaging skin (magnification 200×).

Similarly to the previous report [2], we found UV radiation to cause skin tissue changes, and large quantities of abnormal, tangled, degraded and non-functional fibers were found in the skin of group MC mice. As shown in Figure 1, the epidermal thickness in MC mice was uneven compared to the NC mice. Less destruction of skin structure and the wrinkle formation under the UV radiation were found after treatment with JC and JCH. This result indicated that JC and JCH significantly inhibit wrinkle formation. In group JCH-2, the skin tissue was almost in the same condition as that of the NC group, suggesting an effective protection to the skin tissue against the UV-damage.

#### 3.3.2. The Effects of JC and JCH on Endogenous Collagen of Photoaging Skin

**Figure 2:**
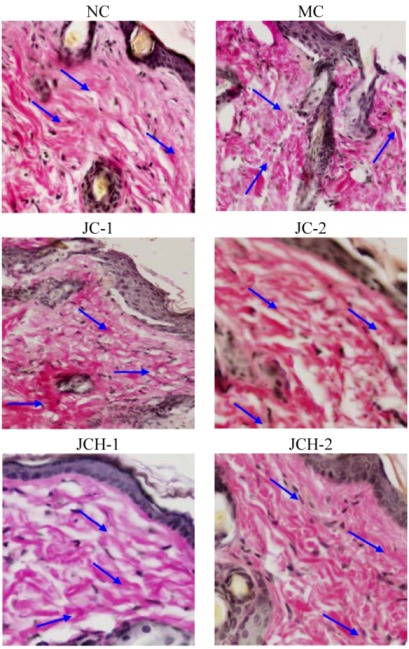
The effect of JC and JCH on endogenous collagen of photoaging skin (magnification 200×); Red: collagen (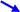).

VG stain is generally used to determine the distribution of skin collagen, which appeared as a red deposit under the microscope. Collagen is the main component of the dermis, constituting 75% of the dry weight of the dermis [2]. The loss of collagenous proteins in irradiated skin is reflected by a decrease in the hydroxyproline content in skin. Our previous study showed that the hydroxyproline content of skin in JC and JCH treated mice was remarkably enhanced [7]. As shown in Figure 2, UV irradiation (MC group) resulted in collagen reduction and tortuosity when compared to the NC group. However, the collagen deposition in JC and JCH groups was significantly increased (Figure 2). In addition, this improvement is more remarkable in JCH group. The collagen improvement is correlated with the hydroxyproline contents increase of the skin tissue. These results indicate that JCH can be used to protect the collagen fibers, and this protective effect is dose-dependent.

#### 3.3.3. The Effects of JC and JCH on Collagen Type I and Collagen Type III of Photoaging Skin

**Figure 3:**
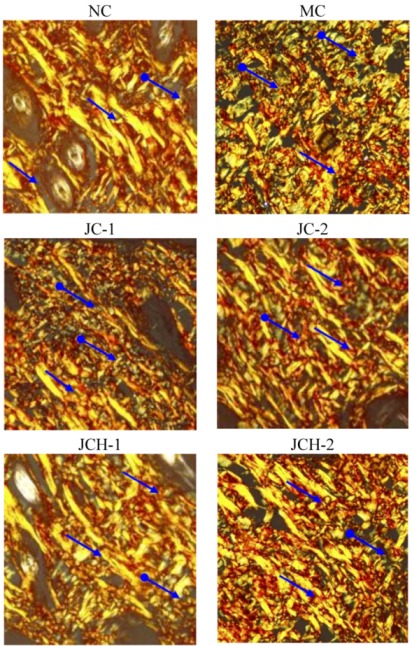
The effect of JC and JCH on collagen type I and collagen type III of photoaging skin (magnification 200×); Yellow-orange: collagen type I (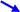), green: collagen type III (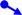).

More than 70% collagen is type I in human skin, which is the most abundant extracellular matrix protein in the human body. It provides the basis for tissue structure and cellular functions [11]. Ultraviolet radiation can increase the type III collagen, while suppressing the type I collagen; consequently, the ratio of type III to type I collagen was increased [12]. Picrosirius red could bind specifically to collagen fibrils of varying diameter and is used to distinguish type I collagen from type III collagen. Under polarized light, collagen fibers can be specifically identified. Type I collagen is stained yellow or orange, whereas type III collagen is stained green [13]. In our study, in the NC group, type I collagen was thick and distributed as a large part of the slices, while type III collagen was hardly seen in the slices (Figure 3). However, in the MC group, the alignment of collagen fibers was disordered and the content of type III collagen increased obviously, which is similar to the previous reports [2,11]. In JC and JCH groups, with the increase of JC and JCH concentration, the intensity of yellow-orange color was also increased, which indicates the increase of collagen deposition (Figure 3). With high concentration of JCH (JCH-2), the distribution of type I and type III was restored to the control level (NC). Therefore, JCH showed the protective effect on photoaging skin, due to its influence on collagen matrix homeostasis. Liang *et al.* (2010) reported that marine collagen hydrolysate was able to promote collagen synthesis, through the activation of Smad signaling pathway and inhibiting collagen degradation by attenuating MMP-1 expression and increasing TIMP-1 expression [14]. Therefore, the effect of JCH on the ratio of type I and type III collagen may be one of the key mechanisms to protect the skin from UV-induced damage.

#### 3.3.4. The Effect of JC and JCH on Elastin Protein Fiber of Photoaging Skin

**Figure 4:**
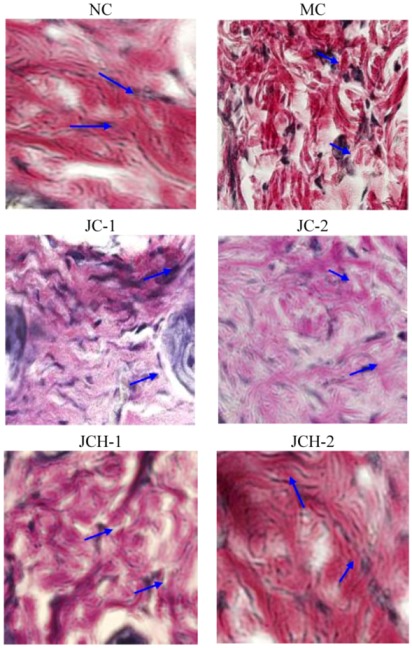
The effects of JC and JCH on elastin protein fiber of photoaging skin (magnification 200×); Blue-black to black: elastic fibres (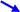).

Elastin protein fibers act as a structural support system and provide the skin with strength and resiliency [15]. The prominent histological feature of photoaging was the result of accumulation of abnormal elastin in the superficial dermis. Elastin accumulation and collagen degradation were prominent characteristics in photodamaged skin. Some researchers demonstrated that decreases in skin elasticity, accompanied by the tortuosity of elastic fibers, were important early events in wrinkle formation [16]. Here we tried to detect the effect of JC and JCH on changes of elastic fiber in skin by Verhoeff-van Gieson stain. As shown in Figure 4, the elastic fibers appeared blue-black to black. In non-irradiated skin, elastic fibers were elongated and slender with a homogeneous distribution (NC group). After exposure to ultraviolet radiation (MC group), elastic fibers in the lower dermis were highly accumulated and appeared short, thickened and twisted (Figure 4). Tortuosity of elastic fibers showed significant difference in the JC and JCH treatments mice, compared to the MC mice. The results confirmed the protective effect of JC and JCH, especially JCH-2, which had no significant difference with the NC group. Ultraviolet irradiation caused the elastic fibers break in the dermis. JCH could significantly protect the elastic fibers in photoaging mice skin. This could be another protective mechanism for the suppression of wrinkle formation.

### 3.4. Thymus Index and Spleen Index

The spleen, the biggest immunity organ, contains a lot of lymphocytes and macrophages, and is the center of the cellular immunity and humoral immunity. The thymus is the most important place in which the T-lymphocytes transform and mature. The atrophy degeneration of thymus means the reduction in the T-lymphocytes, which play a leading role in the function of cellular immune system, and then causes the decline in the cellular immunity, or even suppression [17]. Therefore, thymus index (TI) and spleen index (SI) could be used as the indexes of immunity. In the NC mice, TI was 0.57, while it was significantly decreased to 0.38 in MC group (*p* < 0.05), indicating that ultraviolet radiation had a significant impact on thymus index of mice. However, JC and JCH could significantly increase TI of mice (*p* < 0.05), compared with the MC group. The effect of JCH was significantly stronger than that of JC at the same dose (*p* < 0.05). Even at the low doses, JCH-1 could effectively increase the TI, which had no significant difference with the NC mice (*p* > 0.05). Similarly, spleen index (SI) in the MC mice significantly (*p* < 0.05) decreased to 75.45% of that in NC mice (Table 2). However, JC and JCH treatments could obviously increase the SI of mice (Table 2). At the low doses, the SI in groups JC-1 and JCH-1 was no significant differences to that of the NC group. These results suggested that administration of JC and JCH could enhance the immunity activity of mice. Many studies proved that suberythemal doses of ultraviolet light could affect the immune system in rodents as well as in humans [2,18]. Our study proved that ultraviolet irradiation suppressed the immune system, similar to a previous report [19]. JC and JCH markedly enhanced thymus index and spleen index, thereby protecting against UV-induced damage.

**Table 2:** Effects of JC and JCH on thymus index and spleen index on photoaging mice (*n* = 8).

Group	Thymus Index (mg/g)	Spleen Index (mg/g)
NC	0.57 ± 0.09 a	3.91 ± 0.08 a
MC	0.38 ± 0.10 d	2.95 ± 0.09 b
JC-1	0.47 ± 0.16 c	3.93 ± 0.31 a
JC-2	0.52 ± 0.13 b	3.94 ± 0.48 a
JCH-1	0.56 ± 0.18 a	3.77 ± 0.59 a
JCH-2	0.59 ± 0.15 a	3.82 ± 0.44 a

Different letter indicates significant differences (*p* < 0.05).

## 4. Conclusion

Ultraviolet radiation could cause skin photodamage including immunotoxicity, oxidative damage, decrease of moisture level and histological changes. This study showed that JC and JCH can alleviate the damage induced by UV radiation in dose-dependent manners. The mechanisms of this protection mainly involved enhancing immunity, reducing the loss of moisture, repairing endogenous collagen and elastin protein fibers, and maintaining the ratio of type III to type I collagen. JCH showed much higher bioactivities than JC. Further studies are still on going to elucidate the antiphotoaging mechanisms of JCH.
